# Placental Transfer of Lactate, Glucose and 2-deoxyglucose in Control and Diabetic Wistar Rats

**DOI:** 10.1155/EDR.2001.113

**Published:** 2001

**Authors:** Chris R. Thomas, Beryl B. Oon, Clara Lowy

**Affiliations:** 1 Department of Medicine Guys Kings and St. Thomas School of Medicine St. Thomas' Hospital Lambeth Palace Road London SE1 7EH UK

## Abstract

Placental transfer of lactate, glucose and 2-deoxyglucose was examined employing the *in situ* perfused placenta. Control and streptozotocin induced diabetic Wistar rats were infused with [U^14^C]-glucose and [^3^H]-2-deoxyglucose (2DG). The fetal side of the placenta was perfuseci with a cell free medium and glucose uptake was calculated in the adjacent fetuses. Despite the 5-fold higher maternal plasma glucose concentration in the diabetic dams the calculated fetal glucose metabolic index was not significantly different between the 2 groups. Placental blood flow was reduced in the diabetic animals compared with controls but reduction of transfer of [U^14^C]-glucose and [^3^H]-2-deoxyglucose and endogenously derived [^14^C]-Lactate to the fetal compartment, could not be accounted for by reduced placental blood flow alone. There was no significant net production or uptake of lactate into the perfusion medium that had perfused the fetal side of the placenta in either group. The plasma lactate levels in the fetuses adjacent to the perfused placenta were found to be higher than in the maternal plasma and significantly higher in the fetuses of the diabetic group compared with control group. In this model the *in situ* perfused placenta does not secrete significant quantities of lactate into the fetal compartment in either the control or diabetic group.


Int. Jnl. Experimental Diab. Res., Vol. 2, pp. 113–120
Reprints available directly from the publisher
Photocopying permitted by license only

© 2001 OPA (Overseas Publishers Association) N.V.
Published by license under
the Harwood Academic Publishers imprint,
part of Gordon and Breach Publishing,
a member of the Taylor & Francis Group.
Printed in the U.S.A.

### Placental Transfer of Lactate, Glucose and 2-deoxyglucose in Control and Diabetic Wistar Rats

CHRIS R. THOMAS*, BERYL B. OON and CLARA LOWY

*Department of Medicine, Guys Kings and St. Thomas School of Medicine, St. Thomas' Hospital, Lambeth Palace Road, London SE1 7EH, UK*

*(Received 7 June 2000; In final form 30 April 2001)*

**Placental transfer of lactate, glucose and 2-deoxyglucose was examined employing the *in situ* perfused placenta. Control and streptozotocin induced diabetic Wistar rats were infused with [U-$^{14}$C]-glucose and [$^{3}$H]-2-deoxyglucose (2DG). The fetal side of the placenta was perfused with a cell free medium and glucose uptake was calculated in the adjacent fetuses. Despite the 5-fold higher maternal plasma glucose concentration in the diabetic dams the calculated fetal glucose metabolic index was not significantly different between the 2 groups. Placental blood flow was reduced in the diabetic animals compared with controls but reduction of transfer of [U-$^{14}$C]-glucose and [$^{3}$H]-2-deoxyglucose and endogenously derived [$^{14}$C]-Lactate to the fetal compartment, could not be accounted for by reduced placental blood flow alone. There was no significant net production or uptake of lactate into the perfusion medium that had perfused the fetal side of the placenta in either group. The plasma lactate levels in the fetuses adjacent to the perfused placenta were found to be higher than in the maternal plasma and significantly higher in the fetuses of the diabetic group compared with control group. In this model the *in-situ* perfused placenta does not secrete significant quantities of lactate into the fetal compartment in either the control or diabetic group.**

*Keywords*: Placental transport; Lactate; Glucose; Maternal diabetes; Rats

### INTRODUCTION

Lactate is a three carbon molecule derived from anaerobic metabolism that occurs in most cells. Classically lactate has been considered a product of anaerobic glycolysis or a substrate for gluconeogenesis or glycogenesis in liver. Lactate also has an important role in carbohydrate metabolism by virtue of the rapid equilibrium that exists between lactate and pyruvate, which allows lactate to enter the citric acid cycle. It therefore represents a major energy source.

Under aerobic conditions lactate levels within tissues are usually low due to its rapid turnover rates.[1–4] The exception being in normal mammalian pregnancies where lactate concentrations are higher in fetal than in the maternal circulation, in humans,[5,6] sheep,[7,8] guinea pigs[9] and rats.[10] The elevated levels of this metabolite in the fetal circulation were shown to originate from the placenta in guinea-pigs using an *in vitro* placental preparation.[9] This has also been demonstrated in sheep in *in-vivo* experiments at term,[7,8]

*Corresponding author.

but not at midgestation.[11] This led to the assumption that the elevated fetal plasma lactate levels could be derived from placental anaerobic glycolysis. In diabetic pregnancies fetal plasma lactate concentrations have been reported to be significantly elevated compared with those of non-diabetic pregnancies.[12] Additionally placental glycogen stores are significantly increased[13] compared with those of non-diabetic pregnancies. Thus the higher fetal lactate concentration in the presence of maternal diabetes may be of placental origin, especially as fetal $pO_2$ values of the umbilical vessels from diabetic patients were reported to be within the normal range.[12]

The aim of these studies was to determine whether circulating maternal glucose could be the source of lactate in the fetal circulation of normal and diabetic pregnant rats. This was achieved by examining the transfer of glucose and lactate derived from glucose across the *in-situ* perfused placenta. Fetal plasma concentrations of glucose and lactate as well as tissue glucose uptake were also estimated.

### MATERIALS AND METHODS

### Chemicals

[U-$^{14}$C]-glucose, [$^{3}$H]-2-deoxyglucose (2DG) and Sodium $^{125}$Iodide were obtained from Amersham International plc (Amersham, Bucks.). The anaesthetic Intraval Sodium was obtained from May and Baker (Dagenham, Essex). Ion exchange resins AG 50W 8 × (100–200 mesh, $H^+$ form) and AG 1 × 8 (100–200 mesh, acetate form) used in the separation of lactate from glucose were obtained from Bio-Rad Laboratories (Watford, Herts.) and Optiphase HiSafe scintillant from Wallac UK (Milton Keynes). Lactate dehydrogenase and nicotinamide adenine dinucleotide ($NAD^+$) were from Boehringer Mannheim Biochemica (Lewes, Sussex). Dextran 40 was from Pharmacia LKB Biotechnology (Milton Keynes) and rat insulin standard used in the radioimmunoassay from Nova Biolabs Ltd. (Basingstoke) and the rabbit anti-guinea pig antibodies were from Immunodiagnostics Ltd. (Tyne and Wear). Streptozotocin, bovine serum albumin (Fraction V), D-glucose, antipyrine, perchloric acid, formic acid and all other chemicals used in the enzymatic and radiometric assays were obtained from BDH Chemicals Ltd. (Poole, Dorset) and were of analytical grade.

### Animal Experimentation

Wistar rats (200–250 g; B and K Universal Ltd. Hull) were mated and day 0 of pregnancy was designated as the day a vaginal plaque was noted. On day 4, ten rats were made diabetic by a single intra-peritoneal (i.p.) injection of streptozotocin (40 mg/kg). Rats were maintained on standard breeding rat chow throughout gestation. This diet consisted of 31.5% protein, 43.5% carbohydrate, 4.0% fats, 12.2% dietary fibre and 2.5% supplementations (vitamins, minerals and amino acids). On day 20 of gestation, rats were anaesthetized with Intraval (i.p.) at a dose of 70 mg/kg for controls and 60 mg/kg body weight for diabetic rats. Once anaesthetized, the rat was placed on a thermo-regulated heating pad where the rat body temperature was maintained at 38°C by feedback from a rectal probe.

### Cannulation of Maternal Vessels

Experimentation procedures carried out on the rats were previously described.[14] In brief, the left maternal external jugular vein was cannulated to enable the infusion of radioactive cocktail and antipyrine in saline. The right carotid artery was cannulated for maternal blood sampling and the monitoring of blood pressure *via* a side line connection.

All rats received an initial bolus mixture of 2.4 mCi/8.34 nmol [U-$^{14}$C]-glucose, 4.8 mCi/268 pmol [$^{3}$H]-2-DG and antipyrine in saline followed by a constant infusion rate of 0.036 ml/min 10 min prior to and during the collection of placental perfusion effluent, termed the perfusate. The infusion mixture consisted of 1.2 mCi/4.17 nmol [U-$^{14}$C]-glucose and 2.4 mCi/134 pmol [$^{3}$H]-2-DG in antipyrine-saline solution.

### Placental Perfusion *In Situ*

Once the neck vessels were cannulated and infusion commenced a laparotomy was performed, a fetus was exteriorised to expose the umbilical vein and artery that were then cannulated prior to the removal of the fetus. The placenta was perfused *via* the artery at a rate of 0.5ml/min for 30min, during which 10 three-minute (1.5ml) perfusate fractions were collected from the umbilical vein into chilled pre-weighed tubes. The perfusion fluid consisted of Krebs-Ringer bicarbonate buffer (pH 7.4) supplemented with 30mg/L Dextran 40 and 5g/L bovine serum albumin. D-glucose (5mmol/l for control, 20mmol/l for diabetic animals) and D-lactate (10mmol/l for both control and diabetic animals) were added to the perfusion fluid to mimic fetal glucose and lactate levels (see Tab. II). A measure of a satisfactory experiment was that 96–100% of the inflowing perfusion fluid was recovered after a single passage through the placenta and that the adjacent fetuses were alive until removed from their respective placentae.

### Maternal and Fetal Blood Sampling

During the 30 minute perfusate collection, 6 maternal blood samples (0.6ml) were taken at 6min intervals and divided equally between chilled pre-weighed heparinized test tubes and those containing 10% perchloric acid. At the end of the experiment, the remaining fetuses in utero were sequentially removed. Fetal blood samples were obtained from cut axillary vessels with a heparinized Pasteur pipette and the samples from each litter pooled. A final maternal blood sample was then taken. All plasma samples were aliquoted prior to storage at −20°C.

### Analysis of Samples

Deproteinated samples were kept at −70°C for total lactate and [$^{14}$C]-lactate determinations. These samples were neutralized with 1M KOH prior to analysis. Total lactate determinations were carried out by the method of Engel and Jones.[15] [$^{14}$C]-lactate was separated from the [$^{14}$C]-glucose by a modified method described by Moran *et al.*[16] using microcolumns (0.5cm × 3cm) of ion exchange resins AG 50W 8X (cationic) and AG 1×8 (anionic) in tandem. Recoveries for [$^{14}$C]-glucose, [$^{3}$H]-2-deoxyglucose and [$^{14}$C]-lactate from these microcolumns were between 95–98%. Optiphase Hisafe 3 scintillation fluid was added to the collected eluates and counted for $\beta$-emission in a dual channel programme on the LKB 1219 Rackbeta Spectral liquid scintillation counter. Quencing and spill-over of the high-energy isotope counts into the low-energy counting channel were determined and corrected for by the channel ratio method, using an external standard and two "curves" of $CCl_4$-quenched [$^{3}$H] and [$^{14}$C]n-hexadecane standards.

Maternal and fetal plasma and perfusate were analysed for glucose using the glucose oxidase method (YSI 23 AM analyser, Yellow Springs, OH, USA). Antipyrine was measured in all maternal and perfusate samples by the method of Brodie *et al.*[17] Insulin measurements on maternal and fetal plasma was by an in house radioimmunoassay[18] using an insulin antibody raised in guinea pigs and a rat insulin standard. 2-deoxyglucose-6-phosphate (2DG6P) levels were determined in fetal and placental tissues by the method of Ferre *et al.*[19]

### Calculations

Maternal (M) to perfusate (P) transfer was calculated as the individual ratios of counts in the perfustae (dpm/ml) to that in the maternal plasma (dpm/ml). The values were first averaged from each rat. A grand mean for each group was then calculated. Glucose uptake for fetal and placental tissues were estimated using the glucose metabolic index (GMI) calculated based on the ratio of the concentration of [$^{3}$H]-2DG-6-P within the tissues to the integral of the ratio between

$[^3H]$-2DG to maternal glucose concentration with time, *i.e.*,

$$GMI = \frac{[DG\text{-}6\text{-}P]}{{}^{0}[\text{Maternal DG}]/_{30}[\text{Maternal glucose}]} \cdot dt$$

Statistical significance was tested using Student t-test between variables of the two groups. Values were expressed as means ± standard error of the mean. The specific activities were different for the control and diabetic animals, these are negated by expressing values as perfusate over maternal ratios (P/M).

### RESULTS

Maternal weight, litter size and fetal weight were significantly reduced in the diabetic rats compared with normal rats. The converse was seen with the placentae in the two groups (Tab. I).

The administered dose of streptozotocin increased maternal glycaemia 5 fold (Tab. II) and decreased the maternal plasma level of insulin by 64% (Tab. II). Fetal glucose concentrations in the diabetic group were similarly elevated, however the decrease in fetal plasma insulin level was only 38%. Maternal plasma lactate concentrations were similar in the two groups. Fetal plasma lactate concentrations were significantly higher in the diabetic animals compared with the controls but more striking was the difference in concentration between the maternal and fetal levels in both diabetic and control animals (Tab. II). The placentae were perfused with 10 mmol/l lactate and the perfusate lactate concentation was estimated in each perfusate aliquot. There was a non significant net production of 0.88 ± 0.37 μmol/minute for the control animals and a non significant net uptake of 0.33 ± 0.34 μmol/minute in the diabetic animals. These values were not significantly different from each other. Fetal blood was collected at the end of the experiment from the remaining fetuses when the blood flow to the placenta may have been compromised, possibly resulting in fetal hypoxia and elevating the lactate concentration further.

Antipyrine, a diffusable marker, was used as an indirect measure of maternal placental blood flow. The placental transfer of antipyrine, glucose and lactate was expressed as a ratio of that in the perfusate to that in the simultaneous maternal plasma sample (P:M), (Tab. III). The antipyrine ratio was reduced to 59% in the diabetic group compared to the control group, indicating a decrease in uterine blood flow in the former. The mean P:M transfer ratio of $[^{14}C]$-glucose, $[^{14}C]$-lactate and $[^3H]$-2-DG within the groups were similar but all were substantially reduced in the diabetic group compared

TABLE I Litter size, maternal, fetal and placental weights

| | Control (n = 10) | Diabetic (n = 10) | p value |
|---|---|---|---|
| Litter size | 14.69 ± 0.58 | 12.73 ± 0.67 | 0.05 |
| Maternal Weight (g) | 391.53 ± 4.25 | 343.18 ± 8.64 | 0.0001 |
| Placental Weight (g) | 0.53 ± 0.02 | 0.64 ± 0.03 | 0.01 |
| Fetal Weight (g) | 3.97 ± 0.17 | 3.36 ± 0.19 | 0.05 |

TABLE II Glucose, lactate and insulin plasma concentrations of control (n = 10) and diabetic (n= 10) dams and their fetuses

| | Control (n = 10) | Diabetic (n = 10) | p value |
|---|---|---|---|
| Maternal Plasma Glucose (mmol/l) | 5.1 ± 0.5 | 25.3 ± 2.0 | 0.0000 |
| Fetal Plasma Glucose (mmol/l) | 4.4 ± .6 | 17.1 ± 1.1 | 0.0001 |
| Maternal Plasma Lactate (mmol/l) | 3.1 ± 0.5 | 2.9 ± 0.4 | NS |
| Fetal Plasma Lactate (mmol/l) | 13.3 ± 1.3 | 18.8 ± 1.5 | 0.005 |
| Maternal Insulin (mU/l) | 69.5 ± 8.5 | 24.4 ± 5.6 | 0.005 |
| Fetal Insulin (mU/l) | 43.7 ± 3.2 | 29.8 ± 3.7 | 0.01 |

TABLE III Placental transfer of [$^{14}$C]-glucose, [$^3$H]-2-Deoxyglucose 2, [$^{14}$C]-lactate and antipyrine in control (n = 10) and diabetic (n = 10) dams. The transfer ratios are expressed as the radioactivity in the perfusate (P) to the radioactivity in the simultaneous maternal (M) samples

| | Control (P:M) | Diabetic (P:M) | p value |
|---|---|---|---|
| [$^3$H]-2-Deoxyglucose | 0.433 ± 0.027 | 0.183 ± 0.015 | 0.0001 |
| [$^{14}$C] Glucose | 0.407 ± 0.024 | 0.212 ± 0.04 | 0.005 |
| [$^{14}$C] Lactate | 0.477 ± 0.048 | 0.163 ± 0.018 | 0.0001 |
| Antipyrine | 0.498 ± 0.068 | 0.293 ± 0.024 | 0.01 |

TABLE IV Fetal to maternal plasma ratios of [$^{14}$C]-glucose, [$^3$H]-2-Deoxyglucose and [$^{14}$C]-lactate, determined from the pooled fetal plasma at the end of each experiment

| | Control (F:M) | Diabetic (F:M) | p value |
|---|---|---|---|
| [$^3$H]-2-Deoxyglucose | 0.773 ± 0.006 | 0.843 ± 0.057 | NS |
| [$^{14}$C]Glucose | 0.342 ± 0.057 | 0.731 ± 0.042 | p<0.0001 |
| [$^{14}$C]Lactate | 5.165 ± 0.371 | 4.515 ± 0.333 | NS |

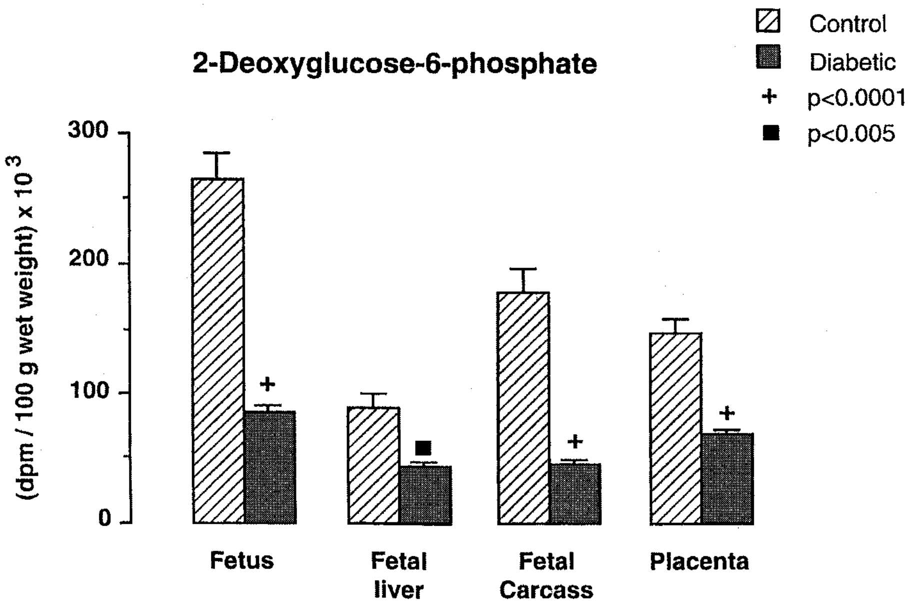

FIGURE 1 2-Deoxyglucose-6-phosphate uptake per 100 grams wet weight in control and diabetic placentas and fetal carcasses.

with values of the control group. When the [$^3$H]-2-DG, [U$^{14}$C]-glucose and [$^{14}$C]-lactate P:M ratios in the diabetic model were corrected for the decrease in antipyrine ratio, the values were 0.310, 0.359, and 0.276 respectively. These values were all lower than the respective control values (Tab. III). Thus the decrease in transfer of the three tracers could not be entirely accounted for by the decrease in the antipyrine ratio.

The transferred [U$^{14}$C]-glucose, [$^3$H]-2-DG and [$^{14}$C]-lactate in the adjacent fetuses were also monitored (Tab. IV) and expressed as the fetal to maternal plasma (F:M) ratios. In fetal plasma [$^3$H]-2-DG and [$^{14}$C]-lactate F:M ratios were similar between the two groups. However, the F:M ratio of [U$^{14}$C]-glucose in the diabetic group was two fold higher compared with controls.

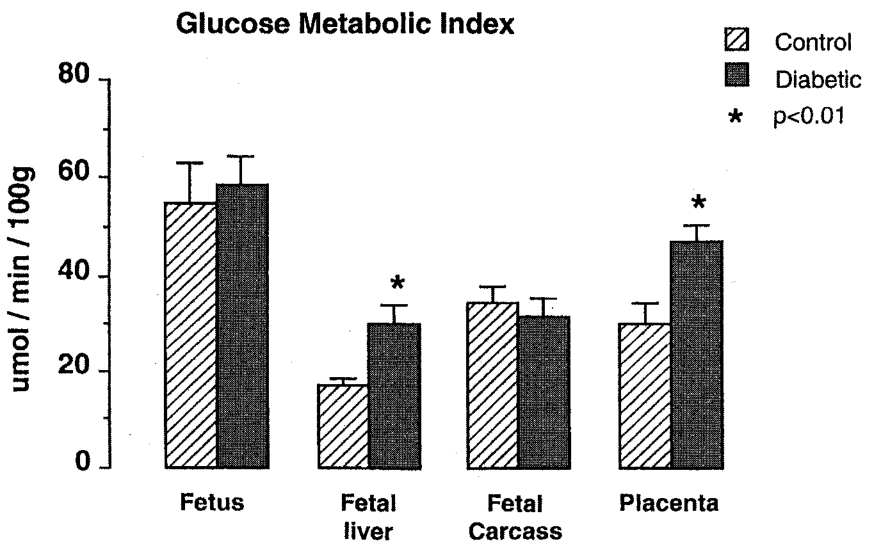

FIGURE 2 The calculated glucose metabolic index of control and diabetic placentas and fetal carcasses lactate.

The quantity of [$^3$H]-2-DG-6-P present in the placentae, whole fetuses and fetal livers of diabetic group was significantly less compared with the controls group ($p<0.05$, $p<0.0005$, $p<0.0005$, respectively; Fig. 1). However, when the GMI was calculated fetal uptake of glucose was not significantly different between control and diabetic animals. The calculated GMI for the fetal livers and placentas were significantly increased in diabetic group compared with the controls (Fig. 2).

### DISCUSSION

The main finding in this rat model was that the elevated fetal lactate concentrations observed in control and diabetic animals were not derived from the placenta, as there was no significant uptake or secretion of lactate from or into the perfusion medium. This was a somewhat unexpected finding. *In vivo* sheep experiments have shown that in mid gestation there is no secretion of lactate by the placenta to the fetal circulation, but at term lactate secretion to the fetus is substantial.[7,11] In our model we only examined placental lactate production and transfer to the fetal compartment. Aldoretta and Hay[20] have examined lactate production derived from glucose in late gestation in sheep under hypoglycaemic and hyperglycaemic states. Less lactate derived from glucose was secreted into the fetus in the hyperglycaemic than in the hypoglycaemic sheep. This may be due to the higher fetal plasma lactate concentration observed in the hyperglycaemic sheep, since direction of lactate transport is determined by the transmembrane lactate concentration.[21] The lactate concentration in the medium perfusing the fetal side of the placenta in our experiments was 10 mmol/l considerably higher than the maternal concentration. This gradient effect may also account for lactate secretion when placental slices are incubated without added lactate in the incubating medium.[22] Approximately 40% of ovine fetal lactate is not derived from glucose and alanine is the likeliest source. The production of [$^{14}$C] lactate from L-[$^{14}$C] alanine has been examined by Palcin *et al.*[22] in pregnant Wistar rats. In their *in vivo* experiments significant quantities of both L-[$^{14}$C] alanine and [$^{14}$C] lactate were present in fetal plasma following the infusion of L-[$^{14}$C] alanine into an external maternal iliac artery. Deamination of the L-[$^{14}$C] alanine may have occurred in either the fetus or the placenta. Fetal plasma lactate levels were 15 mmol/l in their experiments similar to the values in our fetuses adjacent to the perfused placenta. Significant quantities of fetal lactate may thus be derived from fetal rather than from placental alanine deamination. Glycogen levels have been shown to be increased in the diabetic rodent placenta.[13] Lactate production from placental glycogen synthesised from infused universally labelled glucose during the 30-minute experiment would have caused a gradual rise in the P:M [$^{14}$C]-lactate ratio. However, this was not ob-served. The observed placental secretion patterns in different models and at different gestational ages would suggest that there is a control mechanism determining lactate secretion to the fetal and or maternal circulation. Lactate is a universal alternative substrate to glucose for tissues including the brain.[23,24] Thus maintaining the plasma fetal lactate level above the maternal concentration may provide an alternative fetal nutrient.

The observed elevated fetal lactate levels of the diabetic dams compared with controls were not due to maternal to fetal placental transfer since the P:M [$^{14}$C]-lactate ratio, together with the P:M ratio of [U$^{14}$C]-glucose and [$^{3}$H]-2-DG, were significantly lower compared with control animals. Additionally the total maternal plasma lactate levels were similar in the two groups. In this model the elevated fetal lactate values in either group have not been shown to be of placental origin.

The documented presence of very low enzyme activities of the citric-acid-cycle[25] in fetal hepatocytes may account for the presence of elevated fetal lactate concentrations compared with the mother. The significantly higher fetal lactate levels in the presence of maternal diabetes were probably secondary to reduced fetal insulin levels. The combination of the reduced P:M ratio of [U$^{14}$C]-glucose, and a higher F:M ratio in diabetic compared with control animals, suggests that against the background of maternal hyperglycaemia there was reduced fetal glucose uptake. This is supported by the lower deposition of 2-[$^{3}$H]-DG-6-P observed in the fetuses and placentas of diabetic dams. Despite this decrease in 2-[$^{3}$H]-DG-6-P deposition, total glucose uptake from calculated GMI was increased in the placenta. The higher F:M ratio of the specific activities of glucose in the maternal and fetal circulation in the diabetic group ($0.86 \pm 0.05$) compared with controls ($0.41 \pm 0.03$) suggests that the fetuses of the diabetic dams were only able to achieve glucose uptake similar to the control fetuses at hyperglycaemic glucose concentrations. The GMI of the whole fetus in the two groups were similar although fetal liver GMI, an insulin independent tissue, was increased. This could be a protective mechanism whereby the reduced fetal plasma insulin levels limits glucose metabolism in insulin sensitive tissues and prevents excessive somatic growth. This is supported by the observation of reduced fetal myocardial and skeletal muscle insulin sensitive Glut-4 transporter expression in chronically hyperglycaemic sheep.[26] Fetal insulin synthesis would not have been affected by streptozotocin as the drug was administered to the mother prior to the implantation of the conceptus (day 5). Reduced fetal insulin secretion in the diabetic group may, therefore, be a direct result of glucotoxicity, a phenomenon also observed in sheep with chronically elevated maternal plasma glucose levels.[27]

The observed increased diabetic placental glucose uptake is in agreement with Thomas and Lowy[28] but differed in magnitude. This may be related to two factors, the rats in this study were only moderately diabetic compared with the previous study, and they were maintained on a diet optimal for breeding which had been enriched in protein (20.5% w/w) compared to the standard diet used in the previous study.

### CONCLUSION

Placental glucose transport is less efficient in diabetic animals, but is compensated for by the five-fold higher maternal glucose concentration, resulting in an increased delivery of glucose to the fetal compartment. [$^{3}$H]-2DG-6-P deposition was significantly reduced in fetuses of diabetic dams. As the result of the five-fold higher fetal plasma glucose concentration in the diabetic model, the total fetal carcass glucose uptake, as calculated by GMI, was similar in the 2 groups.

This relatively reduced fetal glucose uptake may be due to the low insulin levels in the diabetic animals. The P:M ratios observed for tracer lactate, glucose and 2-deoxyglucose indicate that the placenta will transport lactate. However, the placenta did not secrete significant quantities of lactate in this experimental model. The elevated fetal lactate concentrations are therefore a primary product of fetal metabolism. This metabolic pathway is enhanced in the diabetic animal resulting in higher plasma fetal lactate levels.

### Acknowledgements

This project was supported by the Special Trustees of ST Thomas' Hospital and The British Diabetic Association.

### References

[1] Kreisburg, R. A., Pennington, M. D. and Boshell, B. R. (1970). Lactate turnovers and gluconeogenesis in normal and obese humans. *Diabetes*, **19**, 53–63.
[2] Searle, G. L. and Cavalieri, R. R. (1972). Determination of lactate kinetics in the human analysis of data from single injection *vs.* continuous infusion methods. *Proceedings of the Society for Experimental Biology and Medicine*, **139**(3), 1002–1006.
[3] Freminet, A. and Lecler, L. (1979). Lactate kinetics estimated by single injection and continuous infusion of $^{14}$C-(U)-lactate in rats. *Journal de Physiologie*, **75**(5), 555–557.
[4] Katz, J., Okajima, F., Chenoweth, M. and Dunn, A. (1981). The determination of lactate turnover *in vivo* with $^{3}$H- and $^{14}$C-labelled lactate. The significance of sites of tracer administration and sampling. *Biochemical Journal*, **194**(2), 513–524.
[5] Gilfillan, C. A., Tserng, K. Y. and Kalhan, S. C. (1985). Alanine production by human fetus at term gestation. *Biology of the Neonate*, **47**(3), 141–147.
[6] Bell, J. D., Brown, J. C., Sadler, P. J., Garvie, D., MacLeod, A. F. and Lowy, C. (1989). Maternal and cord blood plasma. Compararative analyses by 1H NMR spectroscopy. *NMR in Biomedicine*, **2**, 61–65.
[7] Burd, L. I., Jones, M. D., Simmons, M. A., Makowski, E. L., Meschia, G. and Battaglia, F. C. (1975). Placental production and fetal utilization of lactate and pyruvate. *Nature*, **254**, 710–711.
[8] Char, V. C. and Creasy, R. K. (1976). Lactate and pyruvate as fetal metabolic substrates. *Pediat. Res.*, **10**, 231–234.
[9] Carstensen, M. H., Leichtweiss, H. P. and Schroeder, H. (1982). The metabolism of the isolated artificially perfused guinea pig placenta. I. Excretion of hydrogen ions, ammonia, carbon dioxide and lactate and the consumption of oxygen and glucose. *J. Perinat. Med.*, **10**, 147–153.
[10] Shambaugh, G. E., Koehler, R. A. and Freinkel, N. (1977). Fetal fuel. II. Contribution of selected carbon fuels to oxidative metabolism in rat conceptus. *Am. J. Physiol.*, **233**, E457–461.
[11] Carter, B. S., Moores, R. R., Bataglia, F. C. and Meschia, G. (1993). Ovine fetal placental lactate exchange and decarboxylation at midgestation. *Am. J. Physiol.*, **264**, E221–225.
[12] Bradley, R. J., Brudenell, J. M. and Nicolaides, K. H. (1991). Fetal acidosis and hyperlacticaemia diagnosed by corcentesis in pregnancies complicated by maternal diabetes mellitus. *Diabetic Medicine*, **8**, 464–468.
[13] Shafrir, E. and Barash, V. (1991). Placental glycogen metabolism in diabetic pregnancy. *Isr. J. Med. Sci.*, **27**, 449–461.
[14] Thomas, C. R., Eriksson, G. L. and Eriksson, U. J. (1990). Effect of maternal diabetes on placental transfer of glucose in rats. *Diabetes*, **32**, 276–282.
[15] Engel, P .C. and Jones, J. B. (1978). Causes and elimination of erratic blanks in enzymatic metabolite assays involving the use of NAD$^+$ in alkaline hydrazine buffers: improved conditions for the assay of l-glutamate, L-lactate, and other metabolites. *Anal. Biochem.*, **88**, 475–84.
[16] Morand, C., Remesy, C. and Demigne, C. (1993). Fatty acids are potent modulators of lactate utilization in isolated hepatocytes from fed rats. *Am. J. Physiol.*, **265**, (5 pt 1), E816–823.
[17] Brodie, B. B., Axelrod, J., Soberman, R. and Levy, B. B. (1949). The estimation of antipyrine in biological materials. *J. Biol. Chem.*, **179**, 25–29.
[18] Sonksen, P. H. (1979). Double antibody technique for the simultaneous assay of insulin and growth hormone. In: Hormones in human blood: detection and assay. (Ed.) Antoniades, H. N., Harvard University press.
[19] Ferre, P., Leturque, A., Burnol, A. F., Penicaud, L. and Girard, J. (1985). A method to quantify glucose utilization *in vivo* in skeletal muscle and white adipose tissue of the anaesthetized rat. *Biochemical Journal*, **228**, 103–110.
[20] Aldoretta, P. W. and Hay, W. W. Jr. (1999). Effect of glucose supply on ovine uteroplacental glucose metabolism. *Am. J. Physiol.*, **277**, (4 pt 2) R947–58.
[21] Kastendieck, E. and Moll, W. (1977). Placental transfer of Lactate and Bicarbonate in the Guinea-Pig. *Pflugers Arch.*, **370**, 165–171.
[22] Palacin, M., Lasuncion, M. A., del Rio, R. M. and Herrera, E. (1985). Placental formation of lactate from transferred L-Alanine and its impairment by aminooxyacetate in the late pregnant rat. *Biochimica et. Biophysica Acta.*, **841**, 90–96.
[23] King, P., Kong, M. F., Parkin, H., MacDonald, I. A., Barbar, C. and Tattersall, R. B. (1998). Intravenous lactate prevents cerebral dysfunction during hypoglycaemia in insulin-dependent diabetic mellitus. *Clinical Science*, **94**, 157–163.
[24] King, P., Parkin, H., MacDonald, I. A., Barbar, C. and Tattersall, R. B. (1997). The effect of intravenous lactate on cerebral function during hypoglycaemia. *Diabetic Medicine*, **14**, 19–28.
[25] Diamant, Y. Z. and Shafrir, E. (1972). Enzymes of carbohydrate and lipid metabolism in the placenta and liver of pregnant rats. *Biochim. Biophys. Acta.*, **279**, 424–430.
[26] Das, V. G., Schroeder, R. E., Hay, W. W. Jr. and Devaskar S. U. (1999). Time dependent and tissue-specific effects of circulating glucose on fetal ovine glucose transporters, *Am. J. Physiol.*, **276** (3 pt 2), R809–17.
[27] Carver, T. D., Anderson, S. M., Aldoretta, P. A., Esler, A. L. and Hay, W. W. Jr. (1995). Glucose suppression of insulin secretion in chronically hyperglycaemic fetal sheep. *Pediatric Research*, **38**(5), 754–62.
[28] Thomas, C. R. and Lowy, C. (1992). Placental transfer and uptake 2-deoxyglucose in control and diabetic rats. *Metabolism*, **41**, 1199–1203.

